# Identification of histidine kinase inhibitors through screening of natural compounds to combat mastitis caused by *Streptococcus agalactiae* in dairy cattle

**DOI:** 10.1186/s13036-023-00378-0

**Published:** 2023-09-26

**Authors:** Rajesh Kumar Pathak, Jun-Mo Kim

**Affiliations:** Department of Animal Science and Technology, Chung-Ang University, Anseong-si, Gyeonggi-do 17546 Republic of Korea

**Keywords:** Cattle, Mastitis, Animal health, Virtual screening, Molecular dynamics, Veterinary drug

## Abstract

**Background:**

Mastitis poses a major threat to dairy farms globally; it results in reduced milk production, increased treatment costs, untimely compromised genetic potential, animal deaths, and economic losses. *Streptococcus agalactiae* is a highly virulent bacteria that cause mastitis. The administration of antibiotics for the treatment of this infection is not advised due to concerns about the emergence of antibiotic resistance and potential adverse effects on human health. Thus, there is a critical need to identify new therapeutic approaches to combat mastitis. One promising target for the development of antibacterial therapies is the transmembrane histidine kinase of bacteria, which plays a key role in signal transduction pathways, secretion systems, virulence, and antibiotic resistance.

**Results:**

In this study, we aimed to identify novel natural compounds that can inhibit transmembrane histidine kinase. To achieve this goal, we conducted a virtual screening of 224,205 natural compounds, selecting the top ten based on their lowest binding energy and favorable protein–ligand interactions. Furthermore, molecular docking of eight selected antibiotics and five histidine kinase inhibitors with transmembrane histidine kinase was performed to evaluate the binding energy with respect to top-screened natural compounds. We also analyzed the ADMET properties of these compounds to assess their drug-likeness. The top two compounds (ZINC000085569031 and ZINC000257435291) and top-screened antibiotics (Tetracycline) that demonstrated a strong binding affinity were subjected to molecular dynamics simulations (100 ns), free energy landscape, and binding free energy calculations using the MM-PBSA method.

**Conclusion:**

Our results suggest that the selected natural compounds have the potential to serve as effective inhibitors of transmembrane histidine kinase and can be utilized for the development of novel antibacterial veterinary medicine for mastitis after further validation through clinical studies.

**Supplementary Information:**

The online version contains supplementary material available at 10.1186/s13036-023-00378-0.

## Introduction

Mastitis is a common disease that affects dairy cattle worldwide. It is an inflammation of the mammary glands caused by bacteria [1]. Mastitis can have a critical impact on milk production, milk quality, and animal welfare as well as cause farmers to suffer reduced profits [1, 2]. It is typically caused by bacteria such as *Staphylococcus aureus*, *Streptococcus* species*,* and *E. coli* [3, 4]. These bacteria can enter the udder through the teat canal, usually during milking or through injuries to the teat or udder [3, 5]. *Streptococcus agalactiae* (*S. agalactiae*) is recognized as the most important pathogen in mastitis with high infectivity [6, 7]. There are multiple virulence factors that contribute to the pathogenicity of a bacterium, which include strong adsorption and anti-phagocytosis mechanisms, as well as immune evasion mechanisms in the case of *S. agalactiae* [6, 8]. *S. agalactiae* is also known to cause severe illnesses in humans, such as sepsis, endocarditis, meningitis, and pneumonia [6, 9].

The symptoms of mastitis include swelling, heat, redness, and pain in the udder [10, 11]. The quality and quantity of milk also decrease in infected cattle [12, 13]. Each instance of mastitis incurs high costs owing to reduced milk production, increased time and effort required for treatment, shortened lifespan of affected animals, high expenses for medical treatment and antibiotics, and the possibility of losing the affected udder quarter or even the lives of cattle [14, 15]. A study conducted by Fukushima et al. looked at 4256 cows. They checked 9663 times when the cows gave birth. Out of these times, 5148 cows (53.3%) had some health issue and needed treatment. Most commonly, the cows had a problem called clinical mastitis, which happened 52.6% of the time, with a rate of 41.6%. The rates for other issues were 21.9% for clinical mastitis, 10.4% for peracute mastitis, 2.9% for metabolic disorders, and 3.2% for peripartum disorders. The number of cows with these issues was 28.0% for clinical mastitis, 13.3% for peracute mastitis, 3.7% for metabolic disorders, and 4.0% for peripartum disorders [16]. The total economic cost of mastitis was calculated to be $444 in the US during the first 30 days of lactation [17, 18]. In farms in Thailand where cows get mastitis, they figured out that it costs about $557 for three months. Out of this, 10.4% was lost because the price of milk went down, and the rest which is 89.6% was because the milk had to be thrown away when they found out a cow had mastitis [19]. In a big milk farming area in Colombia, a study found out that they were losing more than $800.000 every year because of milk losses. This means each cow caused a loss of about $70.30 every year [18, 20]. Therefore, it is globally considered one of the most important and costly diseases in the dairy industry [18].Preventing mastitis in dairy cattle involves the use of antibiotics but antibiotics are not the best solution for treating disease owing to the potential risk of residual antibiotics in milk and the emergence of antibiotic-resistant bacteria [1, 21]. In addition, there are alternative remedies such as herbal and homeopathic treatments that can be used, although they may take longer to provide relief [1, 22]. With the growing population and demand for milk, it is essential to investigate effective ways to cure mastitis with fewer or no adverse effects on hosts, milk consumers, and the environment [23].

Bacterial histidine kinases are characterized as promising drug targets for investigating novel antibacterial agents [24, 25]. It is a part of bacterial two-component systems, involving main signal transduction pathways, and regulating various biological processes including secretion systems, virulence, and antibiotic resistance in bacteria [24]. Therefore, efforts have been made in the present study to model the 3D structure of *Streptococcus agalactiae* histidine kinase from the sequence information for the identification of novel inhibitors against it.

**Fig. 1:**
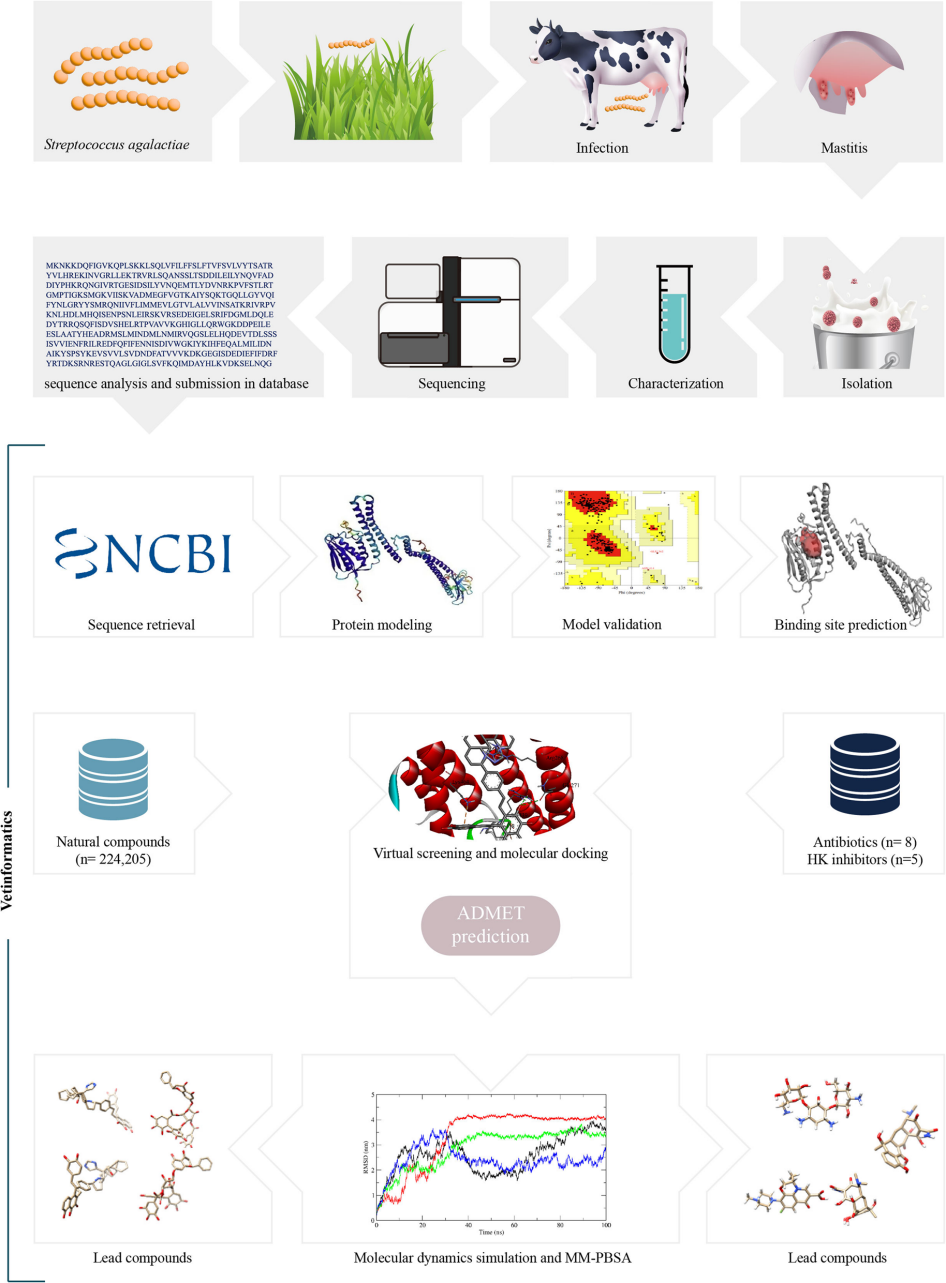
Summary of the work and computational approaches used for identifying natural inhibitors of the transmembrane histidine kinase of S. agalactiae using vetinformatics

In drug discovery programs, natural products play a crucial role in the identification of lead compounds [23, 26–29]. Furthermore, the anti-bacterial potential of natural products is well documented [30, 31]. It was therefore proposed that screening the natural product database against *Streptococcus agalactiae* histidine kinase could serve as the basis for discovering new anti-bacterial lead compounds. In order to find potential new inhibitors of the *Streptococcus agalactiae* histidine kinase, a computational high-throughput virtual screening approach was performed using 224,205 natural compounds from the ZINC database. Furthermore, some antibiotics i.e. Tetracycline, Levofloxacin, Kanamycin, Oxacillin, Benzylpenicillin, Pirlimycin, Erythromycin, and Clindamycin were chosen based on their suitability for treating mastitis in dairy cattle for reference [32]. Molecular docking of selected antibiotics was also performed with *Streptococcus agalactiae* histidine kinase, and results were compared with natural compounds. Besides, the interaction of several reported histidine kinase (HK) inhibitors, namely Waldiomycin, Luteolin, LED209, Maprotiline, and Xanthoangelol B, was also analyzed with S. agalactiae histidine kinase [33]. The results of the top-screened compounds were analyzed and validated using several computational methods, including ADMET profiling, molecular dynamics simulation, Gibbs free energy landscape analysis, and binding energy calculation via MM-PBSA. As a result, novel natural compounds that inhibit histidine kinase were discovered. Finally, a road map for the quick discovery of anti-bacterial agents for the development of veterinary medicine for livestock diseases was presented (Fig. 1).

## Materials and methods

### Sequence retrieval, structural modeling, visualization, validation, binding site prediction and receptor preparation

The amino acid sequence of *Streptococcus agalactiae* transmembrane histidine kinase (Accession number CZT39937.1) was retrieved from the National Center for Biotechnology Information (NCBI) database (https://www.ncbi.nlm.nih.gov/) [34]. AlphaFold2 was used to predict the 3D structure of transmembrane histidine kinase [35, 36]. ModRefiner (https://zhanggroup.org/ModRefiner/) was used to refine the modeled structure [37]. The structural analysis and verification (SAVES) server (https://saves.mbi.ucla.edu/) was used to assess the quality of the modeled structure. Furthermore, the modeled structure was visualized using UCSF Chimera [38] and subjected to the CASTp server (http://sts.bioe.uic.edu/castp/index.html?1ycs) for binding site prediction and analysis [39]. The predicted binding site residues were further confirmed and validated by the COACH server (https://zhanggroup.org/COACH/) [40]. Receptor preparation was conducted using the AutoDock tool [41], which converted the modeled 3D structure into the PDBQT file format from PDB. Charges and polar hydrogens were added during preparation process. Subsequently, a grid box size was created based on the area where binding site residues were predicted.

### Retrieval of antibiotics, histidine kinase inhibitors, natural compounds datasets, and ligand preparation

A literature study directed us to retrieve the 3D structures of eight antibiotics and five histidine kinase inhibitors from the PubChem database (https://pubchem.ncbi.nlm.nih.gov/) in the structure-data file format (SDF) [32, 33]. A subset of 224,205 natural compounds was downloaded from the ZINC database in the sdf file format (https://zinc15.docking.org/substances/subsets/natural-products/) [42]. Ligand preparation was conducted using OpenBabel (https://openbabel.org/wiki/Main_Page) to convert all the compounds into PDBQT (Protein Data Bank (PDB), partial charge (Q), and atom type (T)) files for structure-based virtual screening.

### Structure-based virtual screening and molecular docking

Structure-based virtual screening is a computer-based approach for finding potential new drug-like compounds from a large database. Researchers use virtual screening software to check how well various compounds fit with the binding cavity of a molecular drug target. This helps them to identify molecules that could be used as a starting point in a drug discovery program.

Here, AutoDock Vina was used to perform virtual screening and molecular docking of natural compounds, antibiotics and HK inhibitors with *Streptococcus agalactiae* transmembrane histidine kinase. UCSF Chimera was used to generate protein–ligand complex files of the top-screened natural compounds and other selected compounds i.e. antibiotics and HK inhibitors [38]. The docked protein–ligand complexes were further subjected to Discovery Studio Visualizer (https://discover.3ds.com/discovery-studio-visualizer-download) to analyze the interactions among them, and a protein–ligand interaction diagram was plotted in 2D and 3D. Furthermore, the chemical class of the top two screened natural compounds was identified using the ClassyFire tool (http://classyfire.wishartlab.com/) [43].

### ADMET prediction

ADMET (Absorption, Distribution, Metabolism, Excretion, and Toxicity) properties analysis of the top ten natural compounds was performed by the pkCSM server (https://biosig.lab.uq.edu.au/pkcsm/prediction). The SMILES of the selected compound was retrieved from ZINC database and used as the input file for the pkCSM [44]. The molecular properties (Molecular weight, LogP, Rotatable bonds, Hydrogen bond donor, Hydrogen bond acceptor); absorption (water solubility and intestinal absorption); distribution (Blood–brain Barrier (BBB) permeability, Central nervous system (CNS) permeability); metabolism (CYP2D6 substrate, CYP3A4 substrate, and CYP1A2 inhibitor); excretion (total clearance); and toxicity (AMES toxicity and oral rat acute toxicity) were predicted and analyzed [44].

### Molecular dynamics simulation

The modeled 3D structure of transmembrane histidine kinase, docked complex of top two compounds from the top ten, and the best-screened antibiotic, Tetracycline, were chosen for molecular dynamics simulation and their dynamic behavior determined using GROMACS v2018.1 [45, 46]. The system was prepared as per previous research, the PRODRG server was used to create ligands topology; pdb2gmx and GROMOS96 53a6 force field was utilized to generate the protein topology [1, 27, 28, 47, 48]. A simple point charge water model was used to solvate the system. The topologies for the protein–ligand complexes were generated by merging the protein and ligand topologies and placing them inside cube-shaped boxes [48]. Furthermore, Na^+^ ions were added to maintain the electroneutrality of the system. The energy of the protein and protein–ligand complexes was minimized using the steepest descent minimization algorithm and equilibrated by using NPT and NVT simulations. The molecular dynamics simulation of each system was performed for 100 ns and the coordinates were saved at 2-fs intervals. The trajectories of the protein and protein–ligand complexes were analyzed for structural stability using root-mean-square deviation (RMSD), flexibility using root-mean-square fluctuation (RMSF), compactness using radius of gyration (Rg), ligand-induced solvent-accessible area changes using solvent-accessible surface area (SASA), interactions using hydrogen bonds, and changes in structural movement using principal component analysis (PCA) by GROMACS utilities such as gmx ‘rms’, ‘rmsf’, ‘gyrate’, ‘sasa’, ‘hbond’, ‘covar’, and ‘anaeig’, respectively [27, 28]. Finally, a 2D plotting program Grace (https://plasma-gate.weizmann.ac.il/Grace/) was utilized to analyze and visualize the results.

### Free energy landscape (FEL) and calculation of binding energy through MM-PBSA

The minimum energy states of proteins and protein–ligand complexes were determined using free energy landscape analysis (FEL) with the 'gmx sham' utility in GROMACS. Visualization of the results and production of 2D images were plotted through Grace (https://plasma-gate.weizmann.ac.il/Grace/). Binding free energy computations for selected protein–ligand complexes were conducted using the g_mmpbsa tool, utilizing high-throughput molecular dynamics (MD) simulation data [49].

## Results

### Structural modeling, model assessment, validation, and binding site analysis

AlphaFold2 was utilized to predict 3D model of the transmembrane histidine kinase, and the top-ranked model was selected based on the Local Distance Difference Test (IDDT) score. This model was then refined using the ModRefiner server and assessed for quality using the SAVES server. The resulting PROCHECK analysis, which is based on the Ramachandran plot, showed that 93.5% of the residues were located in the core or most favored regions, 6.0% in additional allowed regions, 0.2% in generously allowed regions, and 0.2% in disallowed regions. These findings suggest that the overall quality of the predicted model was good. Furthermore, CASTp and COACH were used to determine the binding side residues in transmembrane histidine kinase. During the analysis, comparable results were obtained for multiple amino acid residues, including Asp325, Asn395, Lys398, Tyr399, Asp423, Ile428, Tyr441, Arg442, Thr443, Gly455, Leu456, Gly457, Ile458, Gly459, Leu460, Thr484, and Phe486. These residues were predicted to be commonly present in the binding pocket using both tools. The amino acid residues Leu321, Asn324, Asp325, Asn328, Val332, Phe357, Leu360, Arg 361, Gln387, Met390, Ile391, Asp394, Asn395, Ala396, Ile397, Lys398, Tyr399, Asp423, Ile428, Ile436, Phe437, Asp438, Tyr441, Arg442, Thr443, Ser446, Gly455, Leu456, Gly457, Ile458, Gly459, Leu460, Ser461, Val462, Thr484, and Phe486 predicted by CASTp were chosen as binding site residues and utilized during molecular docking studies.

### Investigating transmembrane histidine kinase inhibitors via structure-based virtual screening

Structure-based virtual screening is a computational method that uses the three-dimensional structure of a molecular drug target to identify potential candidate molecules in drug discovery programs. This technique involves the use of molecular docking to predict the binding energy between a set of ligands and receptors. The study demonstrated the use of the ZINC database's natural compounds subset (*n* = 224,205) to perform a virtual screening against transmembrane histidine kinase, in order to identify potential hits. Furthermore, molecular docking of selected antibiotics and histidine kinase (HK) inhibitors was performed as a control to compare the results with those of natural compounds. To identify potential hits for further evaluation, the binding free energy of each compound in the study was assessed. Typically, a protein–ligand complex with low binding energy indicates high binding affinity. As a result, the top ten natural compounds that showed the lowest binding energy with the transmembrane histidine kinase were selected as potential lead compounds (ranging from − 14.4 to − 13.6 kcal/mol). Table 1 lists the ZINC ID, binding free energy, type of interaction, and interacting amino acid residues for these top ten natural compounds. Tables 2 and 3 list the predicted binding energy of the selected antibiotics and HK inhibitors along with interaction type and interacting residues.

**Table 1:** Top ten screened natural compounds, their corresponding binding free energies, types of interactions, and the amino acid residues in the transmembrane histidine kinase involved in interactions

S.N	ZINC ID	Binding energy (Kcal/mol)	Type of interaction	Interacting residues
1.	ZINC000085569031	− 14.4	Conventional hydrogen bond, Pi-cation, Pi-anion, and alkyl bond, Unfavorable positive-positive	Arg268, Gln271, Leu336, Asp438, Arg439, Lys464, Ala469
2.	ZINC000257435291	− 14.2	Attractive charge, conventional hydrogen bond, Carbon hydrogen bond, Unfavorable positive-positive, Unfavorable negative-negative	Tyr265, Arg268, Gln333, Glu337, Gln340, Arg439, Ser461
3.	ZINC000150351499	− 13.9	Attractive charge, conventional hydrogen bond, pi-sigma, pi-alkyl, Unfavorable positive-positive	His290, Ala310, Glu315, Arg318, Glu450
4.	ZINC000253530205	− 13.9	Attractive charge, carbon hydrogen bond, pi-cation, alkyl, pi-alkyl	Arg268, Ile273, Leu336, Asp468, Ala469
5.	ZINC000257787835	− 13.9	Attractive charge, conventional hydrogen bond, pi-cation, pi-anion, pi-sigma, pi-pi t-shaped, pi-alkyl	Arg268, Gln333, Leu336, Asp468, Ala469, His471
6.	ZINC000253529638	-13.8	Attractive charge, conventional hydrogen bond, pi-cation, pi-anion, pi-sigma, pi-pi t-shaped, pi-alkyl	Val286, His290, Ala311, His314, Glu315, Arg318, Glu450
7.	ZINC000150359935	− 13.8	Attractive charge, conventional hydrogen bond, pi-pi t-shaped, alkyl, pi-alkyl, unfavorable positive-positive	Leu336, Phe435, Lys464, Asp468, Ala469, His471
8.	ZINC000276935378	− 13.8	Attractive charge, conventional hydrogen bond, carbon hydrogen bond, pi-cation, unfavorable positive-positive	Glu279, Arg318, Arg447, Leu456
9.	ZINC000253531178	-13.6	Attractive charge, carbon hydrogen bond, alkyl, pi-alkyl	Arg268, Ile273, Leu336, Ala469, Asp468
10.	ZINC000085569053	− 13.6	Attractive charge, conventional hydrogen bond, pi-cation, pi-anion, alkyl, unfavorable positive-positive	Arg268, Gln271, Leu336, Asp438, Arg439, Lys464, Ala469

**Table 2:** Binding free energy of docked antibiotics with transmembrane histidine kinase, types of interactions, and interacting amino acid residues

S.N	Antibiotics	PubChem CID	Binding energy (Kcal/mol)	Type of interactions	Interacting residues
1.	Tetracycline	CID: 54675776	− 8.4	Conventional hydrogen bond, pi-alkyl	His278, Glu279, Phe440, Arg447, Arg442, Leu456
2.	Levofloxacin	CID: 149096	− 8.1	Conventional hydrogen bond, carbon hydrogen bond, halogen (Fluorine), pi-cation, alkyl, pi-alkyl	Glu279, His314, Arg318, Phe440, Arg442, Ala454, Leu456
3.	Kanamycin	CID: 6032	− 7.3	Conventional hydrogen bond	Glu279, His314, Phe440, Arg447, Arg449, Ala454, Gly455,
4.	Oxacillin	CID:6196	− 6.8	Carbon hydrogen bond, pi-cation, alkyl, pi-alkyl	His314, Arg318, Arg442, Arg447, Ala454, Leu456
5.	Benzylpenicillin	CID: 5904	− 6.8	Conventional hydrogen bond, pi-donor hydrogen bond, pi-sigma	Arg268, Gln269, Gln333, Gln465
6.	Pirlimycin	CID:157385	− 6.5	Conventional hydrogen bond, pi-donor hydrogen bond, alkyl, pi-alkyl	Tyr265, Arg268, Leu336, Gln340
7.	Erythromycin	CID:12560	− 6.4	Conventional hydrogen bond, carbon hydrogen bond, Unfavorable acceptor-acceptor	Arg361, Asp394
8.	Clindamycin	CID: 446598	− 6.4	Conventional hydrogen bond, carbon hydrogen bond, pi-sulfur, pi-alkyl	Glu279, His314, Arg318, Arg442, Arg447

**Table 3:** Binding free energy of reported histidine kinase inhibitors with transmembrane histidine kinase, types of interactions, and interacting amino acid residues obtained through molecular docking

SN	HK inhibitors	PubChem CID	Binding energy (Kcal/mol)	Type of interactions	Interacting residues
1	Waldiomycin	CID: 72197554	-8.5	Conventional hydrogen bond, carbon hydrogen bond, Unfavorable acceptor-acceptor, pi-alkyl	Tyr265, Arg268, Gln465, Asp468, Leu472, Lys473, Val474, Asp475, Ile476
2	Luteolin	CID: 5280445	-8.1	Conventional hydrogen bond, Unfavorable donor-donor, Unfavorable acceptor-acceptor, pi-cation, pi-anion, pi-alkyl	Asp325, Ile391, Asp394, Lys398, Gly457
3	LED209	CID: 3421033	-6.8	Attractive charge, Conventional hydrogen bond, pi-anion, pi-sigma, pi-alkyl	Glu279, Arg442, Arg447, Ala454, Gly455, Leu456
4	Maprotiline	CID: 4011	-5.7	pi-anion, pi-alkyl, alkyl	Met390, Asp394
5	Xanthoangelol B	CID: 10409180	-5.2	Conventional hydrogen bond, pi-sigma, pi-alkyl	Ile433, Asp468, Val474, Ile476

### Evaluation and visualization of top-screened docked complexes of natural compounds, antibiotics and HK inhibitors

**Fig. 2:**
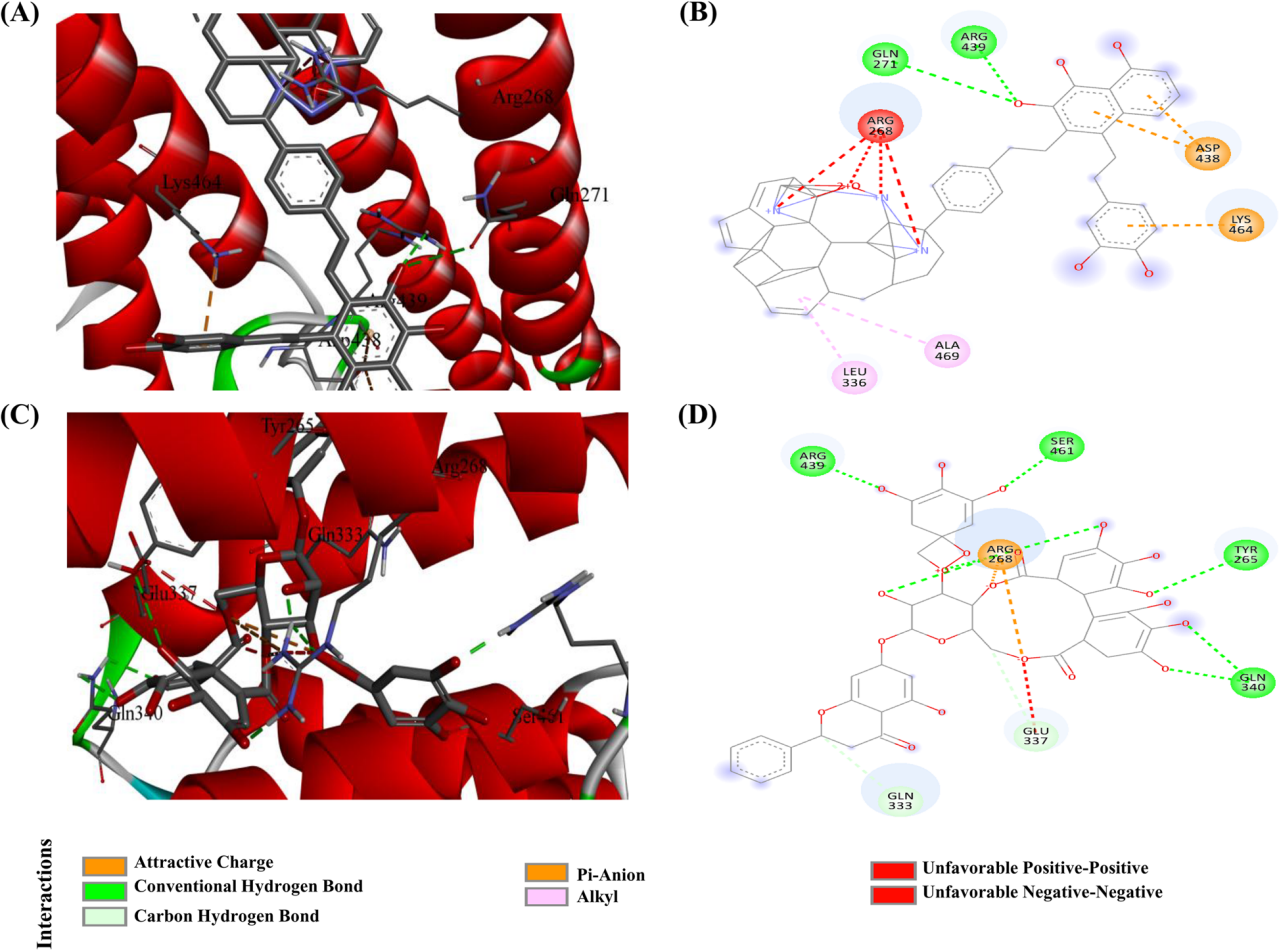
Key amino acid residues that contribute to the protein–ligand interactions of the top two screened natural compounds with the transmembrane histidine kinase of *S. agalactiae* depicted through 3D and 2D representations. **A**-**B** ZINC000085569031, and (**C**-**D**) ZINC000257435291

Based on virtual screening and molecular docking, the binding energy of natural compounds, antibiotics, and HK inhibitors with transmembrane histidine kinase were predicted, and top-screened docked complexes were sorted for further analysis to evaluate their binding nature and inhibitory potential. The top listed natural compound ZINC000085569031 was found to interact with transmembrane histidine kinase amino acid residue Gln271 and Arg439 with conventional hydrogen bonds; alkyl bonds with Leu336 and Ala469; two pi-anion bonds with Asp438 and one pi-cation bond with Lys464; and four unfavorable positive–positive interactions with Arg268 with binding free energy − 14.4 kcal/mol (Fig. 2A-B). Another top listed natural compound, ZINC000257435291, was found to interact with Tyr265, Gln340, Arg439, and Ser461 with conventional hydrogen bonds, forming carbon hydrogen bond with Gln333. Glu337 was found to interact with a carbon hydrogen bond, unfavorable positive–positive and negative–negative; Arg268 was found to involve in protein–ligand interaction with attractive charges and conventional hydrogen bonding with a binding energy 14.2 kcal/mol (Fig. 2C-D). The details of other top-screened compounds are listed in Table 1. However, the top-screened antibiotic Tetracycline was found to interact with His278, Glu279, Phe440, and Arg447 via a conventional hydrogen bond; it interacts with Leu456 via a conventional hydrogen bond and pi-alkyl bond; and Arg442 with pi-alkyl bond and binding energy was predicted as − 8.4 kcal/mol. The details of other analyzed antibiotics and HK inhibitors are listed in Tables 2 and 3, respectively.

### Analysis of drug-likeness properties

ADMET prediction during the initial stages of drug discovery can be highly advantageous in minimizing the occurrence of clinical trial failures. The ADMET properties were predicted by pkCSM. The molecular properties of top-screened natural compounds were predicted. Molecular weights were predicted in the range of 771.972 to 957.39 Dalton; LogP in the range of 0.4633 to 13.2655; Rotatable bonds in the range of 5 to 15; Hydrogen bond acceptor in the range of 7 to 21; and Hydrogen bond donor in the range of 3 to 11. In absorption prediction, water solubility and intestinal absorption are important parameters, which were predicted to be in the range of − 3.397 to − 2.796 and 3.341 to 100 respectively. In distribution, BBB permeability and CNS permeability were predicted to be in the range of − 3.754 to − 0.742 and − 5.633 to − 1.509 respectively. In metabolism, CYP2D6 substrate, CYP3A4 substrate, and CYP1A2 inhibitor parameters were analyzed. The CYP2D6 substrate and CYP1A2 inhibitor were predicted as ‘No’ for all the selected compounds, whereas the CYP3A4 substrate was predicted as ‘No’ for ZINC000257435291, ZINC000257787835, ZINC000253529638, and ZINC000276935378; it was predicted as ‘Yes’ for ZINC000085569031, ZINC000150351499, ZINC000253530205, ZINC000150359935, ZINC000253531178, and ZINC000085569053. In excretion, the total clearance was predicted in the range of − 2.201 to 0.429. In toxicity, the AMES toxicity and oral rat acute toxicity parameter was analyzed. The AMES toxicity was predicted as none for all compounds. Furthermore, the value of oral rat toxicity was predicted in the range of 2.363 to 2.761 (Supplementary Table 1).

### Analyzing the structural and conformational behavior of transmembrane histidine kinase before and after ligand binding

To understand the behavior of transmembrane histidine kinase when bound and unbound to a ligand, we conducted a 100-ns molecular dynamics (MD) simulation. Various parameters, such as RMSD, RMSF, Rg, SASA, H-bond, PCA, FEL, and binding free energy calculations, were used to summarize the obtained results comprehensively.

#### Stability analysis

**Fig. 3:**
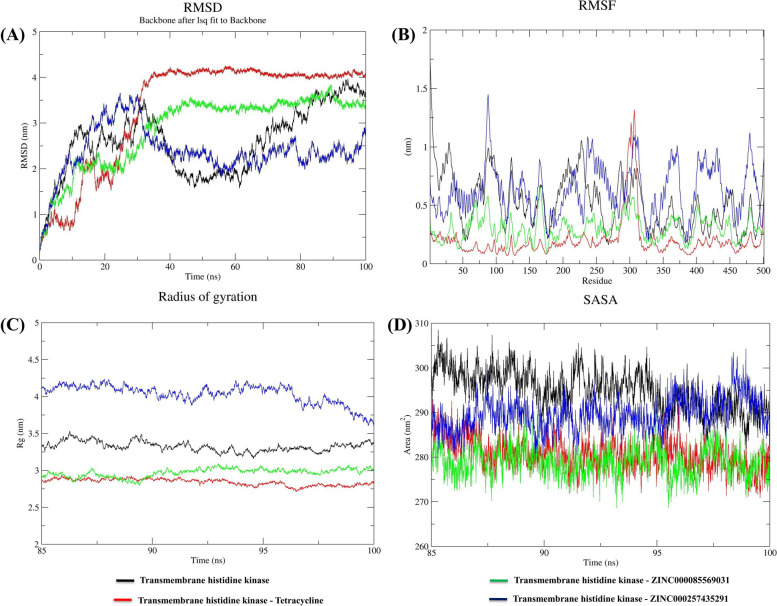
Stability analysis (**A**) RMSD values for the transmembrane histidine kinase-compound complexes. Flexibility analysis (**B**) RMSF values for the transmembrane histidine kinase-compound complexes over the final 15 ns of the simulations. Compactness (**C**) Rg, and Solvent accessible surface area analysis **D** SASA values for the final 15 ns of the simulations

During the molecular dynamics simulation of proteins, the Root Mean Square Deviation (RMSD) was utilized to evaluate the stability of their conformational states. The RMSD value quantifies the deviation between the initial protein structure and the subsequent structures during the simulation, and lower RMSD values indicate higher conformational stability. In this study, the RMSD values were computed for 100 ns, and the backbone c-alpha atoms of the transmembrane histidine kinase and its complexes displayed  minimum fluctuations with lower RMSD values after 85 ns (Fig. 3A). Specifically, the transmembrane histidine kinase had an average RMSD of 2.58 nm, and the RMSD values for its complexes, such as transmembrane histidine kinase-Tetracycline, transmembrane histidine kinase-ZINC000085569031, and transmembrane histidine kinase-ZINC000257435291, were 3.32, 2.87, and 2.38 nm, respectively. Based on the RMSD graph, it was inferred that all complexes reached their equilibrium state after 85 ns, and the final 15-ns trajectories were selected for further analysis.

#### Flexibility analysis

Flexibility is crucial for proteins to maintain their properties, and it can be assessed by analyzing the Root Mean Square Fluctuation (RMSF). In this study, the RMSF analysis was performed on the transmembrane histidine kinase and its complexes during the equilibrated trajectory of the last 15 ns. The results revealed fluctuations in amino acid residues upon the binding of ligands(Fig. 3B). The average RMSF value of the transmembrane histidine kinase was found to be 0.52 nm. Furthermore, the RMSF values of the transmembrane histidine kinase in complexes with Tetracycline, ZINC000085569031, and ZINC000257435291 were 0.20 nm, 0.30 nm, and 0.62 nm, respectively.

#### Compactness analysis

To investigate the compactness, stability, and folding of protein structures, it is possible to analyze Rg values over time. In this work, the Rg values were examined for transmembrane histidine kinase and its complexes, with the aim of assessing their structural compactness. Specifically, the Rg values for the transmembrane histidine kinase, transmembrane histidine kinase-Tetracycline, transmembrane histidine kinase-ZINC000085569031, and transmembrane histidine kinase-ZINC000257435291 systems were calculated and plotted based on the final 15 ns MD trajectories (Fig. 3C). The average Rg values for these systems were 3.32 nm, 2.84 nm, 2.96 nm, and 4.03 nm, respectively. The findings indicate that the transmembrane histidine kinase-Tetracycline complex demonstrates a more compact structure compared to the other complexes.

#### Solvent accessible surface area analysis

SASA analysis was conducted from the final 15 ns of simulation; we determined the changes in the solvent-accessible area induced by ligands. The average SASA values for transmembrane histidine kinase, transmembrane histidine kinase-Tetracycline, transmembrane histidine kinase-ZINC000085569031, and transmembrane histidine kinase-ZINC000257435291 were computed as 295.0, 280.6, 278.8, and 289.6 nm^2^, respectively. Notably, the SASA value for the transmembrane histidine kinase-ZINC000257435291 complex was higher than that of the other  complexes. Furthermore, all systems exhibited a similar pattern, indicating that each compound induced relatively minor changes upon binding (Fig. 3D).

#### Hydrogen bond analysis

**Fig. 4:**
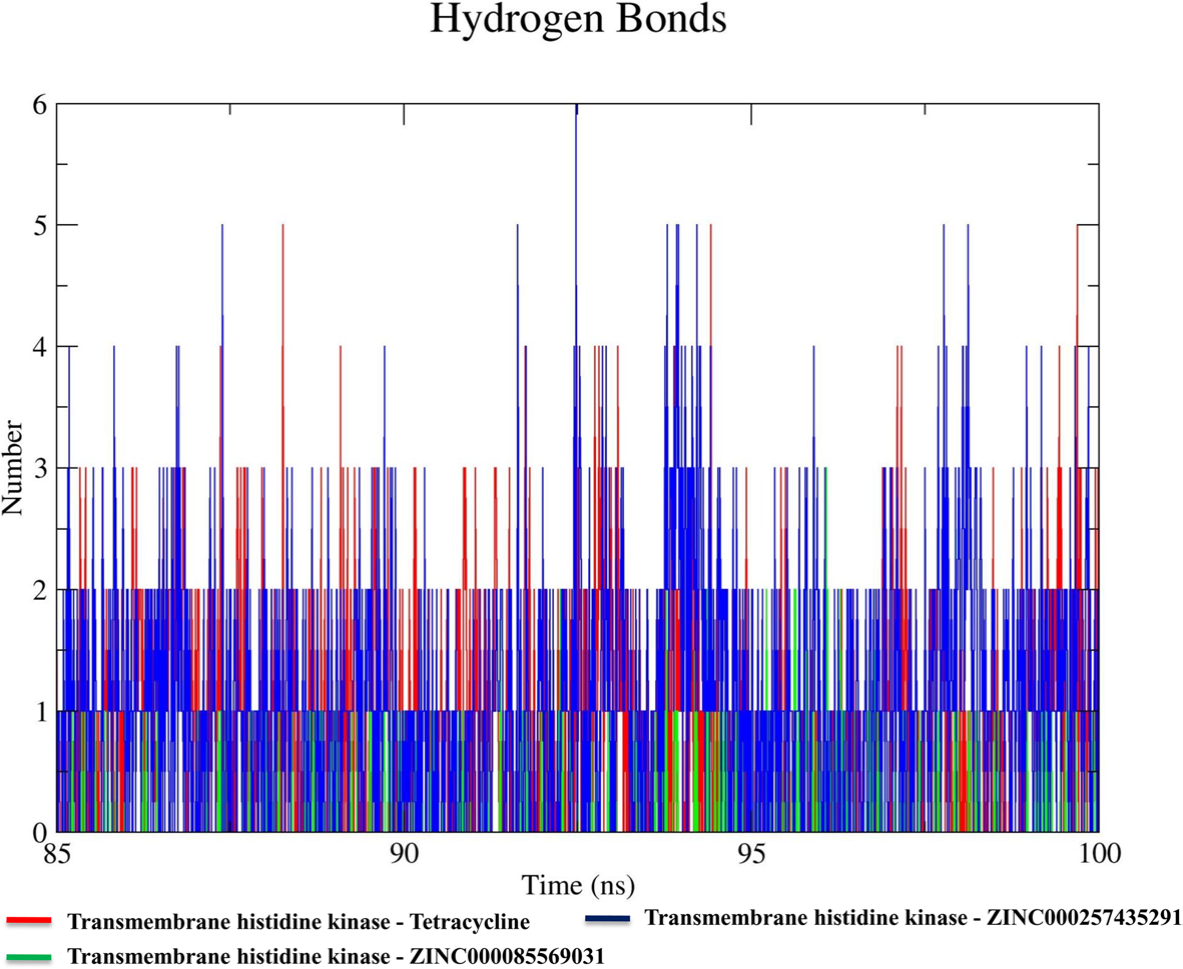
Number of hydrogen bonds in each complex was analyzed based on the data obtained from the last 15 ns of the simulations

The hydrogen bond is the most crucial binding force that stabilizes protein–ligand interactions. To quantify the hydrogen bonds formed during the interaction between natural compounds and the transmembrane histidine kinase target, we conducted an analysis. Our results showed that the transmembrane histidine kinase-ZINC000257435291 complex had the highest number of hydrogen bonds (0–6) compared to the other estimated complexes. The transmembrane histidine kinase-ZINC000085569031 complex formed 0–3 hydrogen bonds, while the reference molecule transmembrane histidine kinase-Tetracycline complex formed 0–5 hydrogen bonds during the final 15 ns. Therefore, these compounds interacted with the transmembrane histidine kinase to produce stable complexes through the formation of hydrogen bonds (Fig. 4).

#### Principal component analysis

**Fig. 5:**
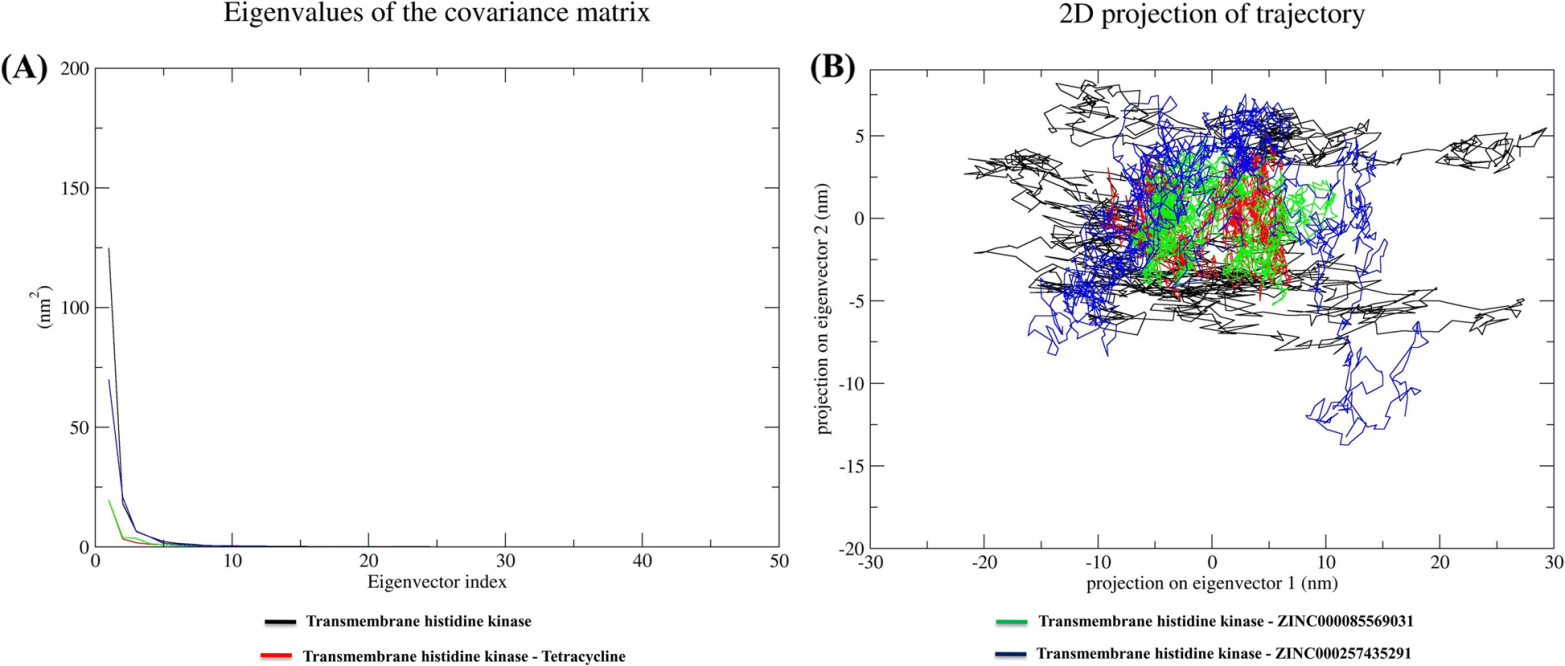
Principal component analysis. **A** Eigenvalues derived from the final 15 ns of each simulation and used for PCA analysis depicted eigenvalues vs. the first fifty eigenvectors. **B** The first two eigenvectors depicted the transmembrane histidine kinase motion in space for all the systems

Principal component analysis (PCA) was employed to detect significant conformational changes upon ligand binding. The protein's global motion is primarily influenced by the first few eigenvectors, and therefore, the first 50 eigenvectors were examined to identify structural variations in the movement. The initial five eigenvectors were used to calculate the percentage of correlated motions, which provided a clear understanding of the changes caused by ligand binding. The correlated motions of the transmembrane histidine kinase, transmembrane histidine kinase-Tetracycline, transmembrane histidine kinase-ZINC000085569031, and transmembrane histidine kinase-ZINC000257435291 complexes were found to be 95.09%, 83.92%, 87.03%, and 94.77%, respectively, with the lowest motion observed in the transmembrane histidine kinase-Tetracycline complex (Fig. 5A).

The first few eigenvectors reflect the protein's overall dynamics, as illustrated in the figure, leading to the selection of the first two eigenvectors and their plotting in phase space. The transmembrane histidine kinase-Tetracycline, and transmembrane histidine kinase-ZINC000085569031 complexes clustered together and exhibited higher stability, with low correlated motions compared to the transmembrane histidine kinase and transmembrane histidine kinase-ZINC000257435291 (Fig. 5B).

### Gibbs free energy landscape analysis

**Fig. 6:**
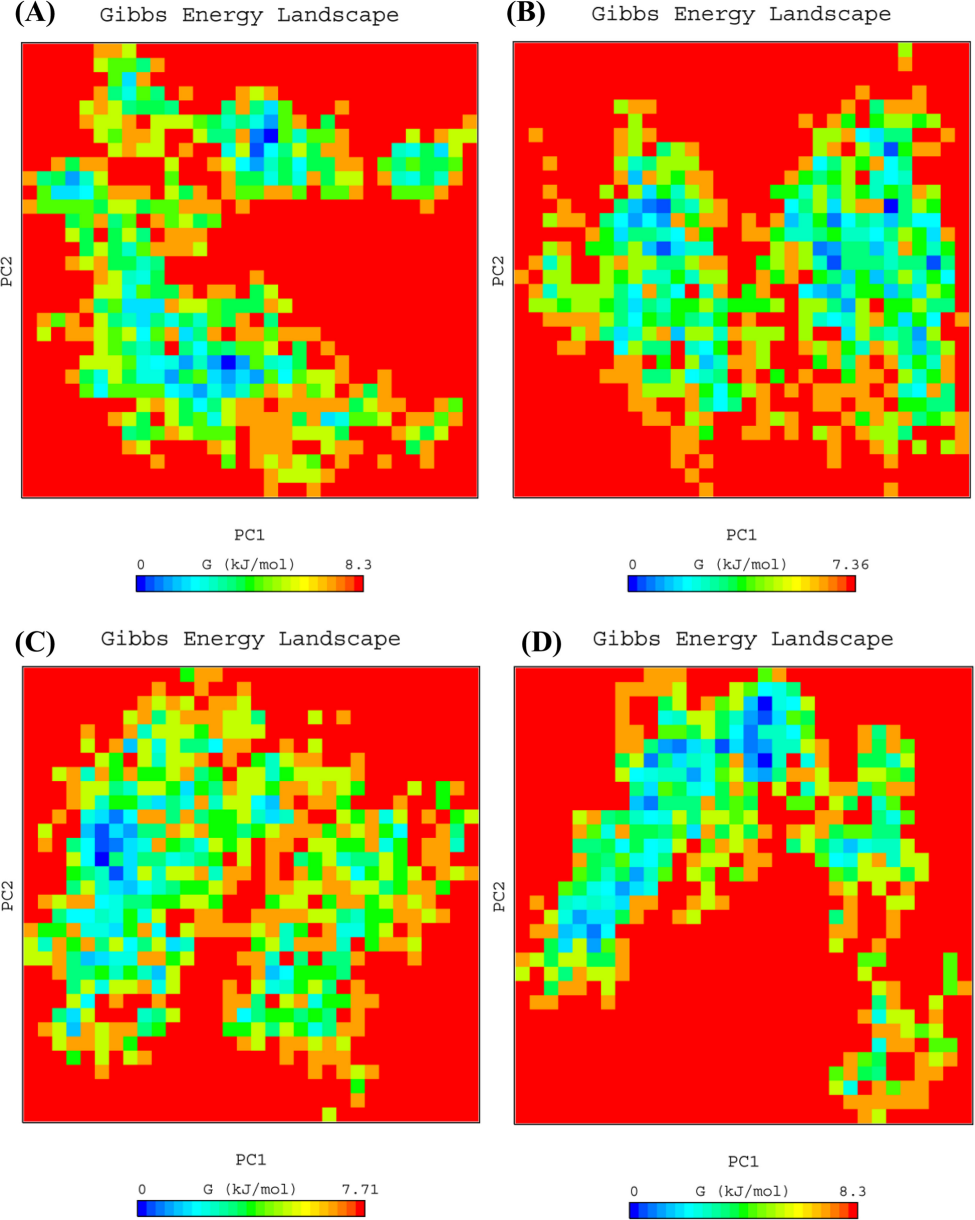
Gibbs free energy landscape has been depicted using a color-coded illustration, which is plotted based on PC1 and PC2. The contour map represents various energy states, with the lower energy systems represented by a deeper blue color. **A** Transmembrane histidine kinase (**B**) Transmembrane histidine kinase-Tetracycline (**C**) Transmembrane histidine kinase-ZINC000085569031,and (**D**) Transmembrane histidine kinase-ZINC000257435291

Gibbs free energy landscape (FEL) calculations were carried out using the first two principal components, and the resulting FEL for all the systems is presented in Fig. 6. The color bar in the figure represents the Gibbs free energies (in units of kJ mol^−1^), ranging from the lowest energy (shown in blue) to the highest energy (represented by the red color) conformational states. The analysis revealed that the transmembrane histidine kinase-Tetracycline (0–7.36 kJ mol^−1^) and transmembrane histidine kinase-ZINC000085569031 (0–7.71 kJ mol^−1^) systems exhibited enriched energy minima indicated by the larger blue area. These systems were found to form a stable cluster compared to transmembrane histidine kinase (0–8.3 kJ mol^−1^) and transmembrane histidine kinase-ZINC000257435291 (0–8.3 kJ mol^−1^) systems. Based on the FEL analysis, all the systems gained minimum energy corresponding to the most stable conformations. Therefore, this study demonstrates that the FEL analysis provides insights into the stability and conformational behavior of the protein–ligand complexes.

### Calculation of binding free energy and residual binding energy analysis

**Fig. 7:**
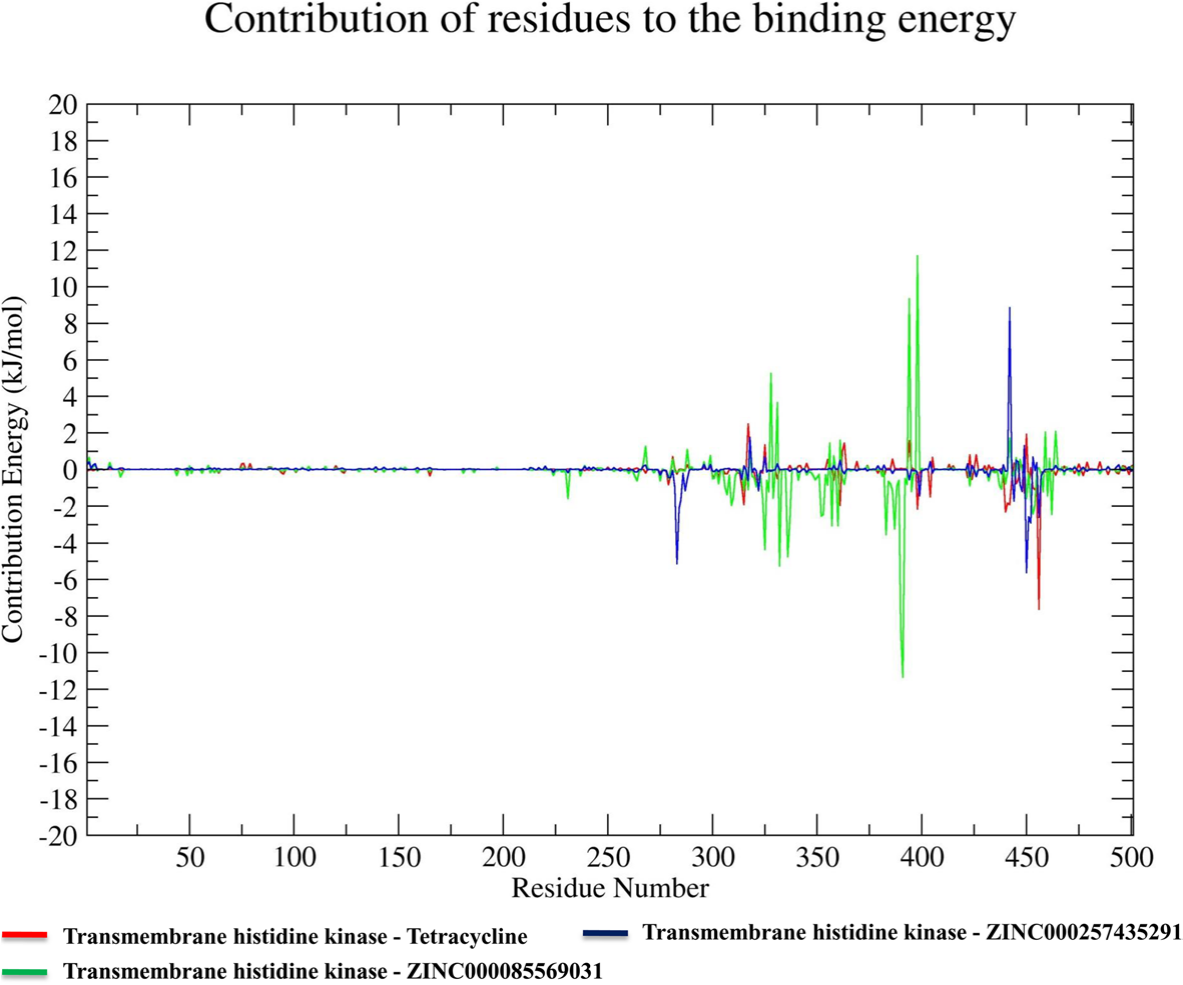
Amino acid residues in transmembrane histidine kinase that participate in the interactions with reference Tetracycline and natural compounds ZINC000085569031 and ZINC000257435291

To evaluate the strength of the binding affinities between simulated complexes, an MM-PBSA method was utilized to calculate their binding free energy. The binding free energies were determined based on the last 10 ns of the molecular dynamics simulation trajectories. The resulting values for the transmembrane histidine kinase-Tetracycline, transmembrane histidine kinase-ZINC000085569031, and transmembrane histidine kinase-ZINC000257435291 complexes were − 67.697, − 230.136, and − 125.662 kJ mol^−1^, respectively. The calculated values for the various energy contributions, such as van der Waals, electrostatic, polar solvation, SASA, and binding free energies are presented in Table 4. Furthermore, a residual binding energy analysis was performed to identify the key amino acid residues that are important for ligand binding. All of the selected compounds were observed to significantly interact with amino acid residues of the transmembrane histidine kinase, which suggests that there is potential for transmembrane histidine kinase inhibitors. Furthermore, amino acid residues from positions 280 to 470 were found to be more involved in the protein–ligand interactions for inhibition of transmembrane histidine kinase activities (Fig. 7).

**Table 4:** Affinities of top-two screened natural compounds and reference Tetracycline with transmembrane histidine kinase (van der Waals and electrostatic forces, polar solvation, SASA, and binding free energy in kJ mol-1)

Compound name	van der Waals energy	Electrostatic energy	Polar solvation energy	SASA energy	Binding energy
Tetracycline	-119.300 ± 14.228	-33.794 ± 17.615	97.934 ± 27.452	-12.538 ± 1.377	-67.697 ± 12.920
ZINC000085569031	-314.040 ± 17.840	-5.482 ± 4.238	118.041 ± 17.386	-28.655 ± 1.725	-230.136 ± 20.754
ZINC000257435291	-213.989 ± 20.278	-23.553 ± 13.282	133.304 ± 38.984	-21.424 ± 3.563	-125.662 ± 42.584

## Discussion

In this study, we demonstrated the tremendous potential of computational approaches to identify lead compounds for development of antibacterial drugs against mastitis utilizing structure-based virtual screening, ADMET prediction, molecular dynamics simulation, Gibbs free energy, and binding energy calculations [50, 51]. Mastitis is a prevalent infectious disease that affects dairy cattle worldwide, and it is a leading cause of reduced milk production efficiency. The condition is a matter of considerable concern within the dairy industry due to its association with adverse behavioral and heightened stress responses in the affected cattle.It can have major financial implications, ultimately leading to substantial economic losses in the industry [1, 52]. The use of antibiotics for treating mastitis is limited owing to the presence of harmful residual levels in milk, which can have negative effects on human health. Furthermore, the continuous use of antibiotics can lead to the development of antibiotic-resistant bacteria [21, 53]. While herbal and homeopathic remedies can be effective against the disease, they often require a longer time to show therapeutic effects [22]. Thus, identifying new antibacterial compounds from natural sources is a promising strategy for protecting cattle from mastitis. However, traditional drug discovery methods can be time-consuming and expensive. A vetinformatics-guided approach can provide cost-effective and timely solutions by using computational resources such as tools and databases to screen natural compound databases for novel compounds that can act as effective drugs against mastitis [23, 28]. Therefore, transmembrane histidine kinases, which regulate various processes such as growth, vitality, antibiotic resistance, and virulence, have been considered as promising targets for the discovery of natural compound inhibitors [33]. There are several classes of histidine kinase inhibitors being investigated, including isothiazolidones, benzoxazines, imidazolium salts, salicylanilides, benzimidazoles, thiophenes, thiazolidiones, and other derivatives [33, 54]. However, they suffer from poor bioavailability, antimicrobial resistance and a lack of selectivity [55]. A recent study based on molecular docking and simulations predicted the interaction of waldiomycin and its methyl ester analog with *S. aureus* histidine kinase [55]. Therefore, due to the significance of histidine kinases in antibacterial drug discovery, more research is needed to identify new and potent inhibitors using structural bioinformatics approaches [25, 55].

From the screening of natural compounds database against transmembrane histidine kinase, the top ten compounds were selected for further analysis using various computational approaches [56]. Some selected antibiotics and HK inhibitors were also docked to the transmembrane histidine kinase and the results compared with natural compounds [32]. Based on our analysis, natural compounds showed better binding affinity as compared to chosen antibiotics and HK inhibitors. Therefore, ADMET profiling of top ten screened natural compounds was analyzed and predicted as drug-like candidates [44]. Although the top-screened compound does not follow Lipinski's Rule of 5, it may still have potential for use as a drug. It is important to note that there are many drug molecules that have been approved for use despite not following the rule of 5 [57, 58]. Therefore, the identified molecules can be considered for further analysis and evaluation. The overall stability of the top two docked complexes, i.e., transmembrane histidine kinase-ZINC000085569031 and transmembrane histidine kinase-ZINC000257435291 along with reference transmembrane histidine kinase-Tetracycline complex were through molecular dynamics simulation. Molecular dynamics simulation is a powerful computational method and has tremendous potential to dissect the behavior and interacting nature of drug and target [59, 60]. The stability analysis using RMSD demonstrated that all systems were equilibrated after 85 ns, indicating significant interactions between the predicted natural lead compounds and transmembrane histidine kinase. Consequently, the final 15 ns trajectories were evaluated to investigate additional parameters using RMSF, Rg, SASA, H-bonds, PCA, and FEL to comprehend the properties of selected compounds [50]. The results indicated that the binding of the natural compounds not only modifies the transmembrane histidine kinase conformation but also alters the necessary dynamics for inhibition.

The present study conducted further analysis on the binding affinity of natural compounds towards the transmembrane histidine kinase through binding free energy and residual binding energy calculations using the MM-PBSA method [49, 61]. This calculation is a well-accepted approach for determining the binding free energy of protein–ligand complex obtained from MD simulation results [61]. Measuring each natural compound’s binding affinity, which directly relates to its potency, enabled to determine the strength of the binding contact between each selected compound i.e. ZINC000085569031, ZINC000257435291, reference Tetracycline, and transmembrane histidine kinase, which is crucial in drug discovery. Furthermore, favorable reactions have negative binding energy, which enhances interactions, while high binding energy of protein–ligand complexes is associated with lower binding affinity [62]. Through the MM-PBSA and residual binding energy calculations, the study concluded that analyzed complexes were energetically stable. These findings suggest that ZINC000085569031 and ZINC000257435291 could be potential leads for the development of antibacterial therapeutics against mastitis. The suggested compounds belong to the class of quinolines and derivatives, and flavonoids, respectively predicted and analyzed by the ClassyFire tool [43]. These classes of compounds are also reported as inhibitors for certain kinases, such as tyrosine kinases and other protein kinases [63, 64]. Additionally, luteolin, a reported histidine kinase inhibitor, also belongs to the flavonoid class [33, 64].

The use of computational resources and vetinformatics can help in identifying potential veterinary drugs by examining natural compound databases against drug targets [23]. This approach can decrease the time and cost associated with experimentation and enhance research output [59, 60]. A majority of medicines available today are sourced from nature or natural chemicals [26, 65]. Furthermore, the effectiveness of natural compounds in fighting bacterial pathogens is described in scientific literature [65, 66]. Therefore, the results of the present study can be utilized in developing antibacterial therapeutics that target transmembrane histidine kinase of *Streptococcus agalactiae* for treating mastitis in dairy cattle.

## Conclusion

*Streptococcus agalactiae* infections pose a major threat to the health of dairy cattle and dairy industry globally. The use of antibiotics is limited owing to major concerns associated with human health and developing antimicrobial resistance in the bacteria. Moreover, several classes of identified histidine kinase inhibitors suffer from drug resistance, poor bioavailability, and a lack of selectivity. Therefore, it is urgent to investigate new and effective antibacterial therapeutics. In the present study, several computational techniques were utilized to identify natural compounds capable of inhibiting the transmembrane histidine kinase of *Streptococcus agalactiae*. The results of the present study suggest that ZINC000085569031 and ZINC000257435291 have the potential to be developed as antibacterial veterinary drugs for mastitis. In the future, the antibacterial activity of these compounds can be optimized to enhance their potential, and clinical studies can be conducted to develop new veterinary medicine.

### Supplementary Information

**Additional file 1:** **Supplementary Table 1.** Predicted ADMET properties of the top ten screened natural compounds.

## Data Availability

All data generated or analyzed during this study are included in the manuscript.

## Abbreviations

MD: Molecular dynamics;
RMSD: Root-mean-square deviation
RMSF: Root-mean-square fluctuation
Rg: Radius of gyration
SASA: Solvent-accessible surface area
HBs: Hydrogen bonds
PCA: Principal component analysis
FEL: Free energy landscape
MM-PBSA: Molecular mechanics Poisson–Boltzmann surface area
