# Supplementary material for: Identification of histidine kinase inhibitors through screening of natural compounds to combat mastitis caused by Streptococcus agalactiae in dairy cattle
Source: J Biol Eng. 2023 Sep 26;17:59. doi: 10.1186/s13036-023-00378-0 (PMC10523694; doi:10.1186/s13036-023-00378-0)
Supplement: Supplementary file 1 — Additional file 1: Supplementary Table 1. Predicted ADMET properties of the top ten screened natural compounds. [file 13036_2023_378_MOESM1_ESM.doc]

**Supplementary Table 1.** Predicted ADMET properties of the top ten screened natural compounds.

| **Property** | **Parameter** | **ZINC000085569031** | **ZINC000257435291** | **ZINC000150351499** | **ZINC000253530205** | **ZINC000257787835** | **ZINC000253529638** | **ZINC000150359935** | **ZINC000276935378** | **ZINC000253531178** | **ZINC000085569053** |
| --- | --- | --- | --- | --- | --- | --- | --- | --- | --- | --- | --- |
| Molecular properties | MW | 815.003 | 872.697 | 932.936 | 771.972 | 899.081 | 955.145 | 957.39 | 883.082 | 771.972 | 790.981 |
|  | LogP | 9.37729 | 3.2023 | 3.29148 | 3.532 | 0.4633 | 1.9161 | 13.2655 | 1.8387 | 3.532 | 8.89619 |
|  | Rotatable bonds | 7 | 5 | 13 | 6 | 9 | 10 | 15 | 9 | 6 | 9 |
|  | HB acceptor | 9 | 21 | 17 | 10 | 17 | 17 | 7 | 15 | 10 | 9 |
|  | HB donor | 7 | 11 | 9 | 4 | 9 | 9 | 3 | 9 | 4 | 7 |
| Absorption | Water solubility(log mol/L) | −2.892 | −2.892 | −2.892 | −3.397 | −2.796 | −2.874 | −2.892 | −2.868 | −3.397 | −2.892 |
|  | Intestinal absorption(% Absorbed) | 100 | 57.76 | 21.712 | 100 | 29.595 | 11.264 | 100 | 3.341 | 100 | 96.353 |
| Distribution | BBB permeability(log BB) | −1.131 | −3.754 | −2.269 | −0.742 | −1.756 | −1.691 | −1.115 | −1.518 | −0.742 | −1.218 |
|  | CNS permeability(log PS) | −2.721 | −5.633 | −4.835 | −3.228 | −4.034 | −4.557 | −1.509 | −3.707 | −3.228 | −2.741 |
| Metabolism | CYP2D6 substrate(Yes/No) | No | No | No | No | No | No | No | No | No | No |
|  | CYP3A4 substrate(Yes/No) | Yes | No | Yes | Yes | No | No | Yes | No | Yes | Yes |
|  | CYP1A2 inhibitor(Yes/No) | No | No | No | No | No | No | No | No | No | No |
| Excretion | Total Clearance(log ml/min/kg) | 0.012 | −2.201 | −1.255 | 0.429 | 0.132 | −0.065 | −0.637 | 0.291 | 0.429 | 0.261 |
| Toxicity | AMES toxicity(Yes/No) | No | No | No | No | No | No | No | No | No | No |
|  | Oral Rat Acute Toxicity (LD50)(mol/kg) | 2.444 | 2.48 | 2.49 | 2.535 | 2.761 | 2.574 | 2.468 | 2.609 | 2.535 | 2.363 |
|  | Oral Rat Chronic Toxicity (LOAEL)(log mg/kg_bw/day) | 5.033 | 9.249 | 6.116 | 2.141 | 5.639 | 5.307 | 4.17 | 6.542 | 2.141 | 4.718 |
